# Trends in elevated waist-to-height ratio and waist circumference in U.S. adults and their associations with cardiometabolic diseases and cancer, 1999–2018

**DOI:** 10.3389/fnut.2023.1124468

**Published:** 2023-04-11

**Authors:** Bo Yang, Jingli Yang, Martin Ming-him Wong, Juwel Rana, Qinghua Yang, Vicky Chan, Moyukh Shabon Khan, Aimin Yang, Kenneth Lo

**Affiliations:** ^1^Department of Epidemiology and Center for Global Cardiometabolic Health, School of Public Health, Brown University, Providence, RI, United States; ^2^College of Earth and Environmental Sciences, Lanzhou University, Lanzhou, China; ^3^School of Professional and Continuing Education, The University of Hong Kong, Pok Fu Lam, Hong Kong SAR, China; ^4^Department of Epidemiology, Biostatistics and Occupational Health, School of Medicine and Health Sciences, McGill University, Montreal, QC, Canada; ^5^Department of Public Health, School of Health and Life Sciences, North South University, Dhaka, Bangladesh; ^6^Department of Nephrology, Peking University International Hospital, Beijing, China; ^7^Department of Food Science and Nutrition, The Hong Kong Polytechnic University, Kowloon, Hong Kong SAR, China; ^8^Department of Medicine and Therapeutics, The Chinese University of Hong Kong, Shatin, Hong Kong SAR, China; ^9^Research Institute for Smart Ageing, The Hong Kong Polytechnic University, Kowloon, Hong Kong SAR, China

**Keywords:** waist-to-height ratio, waist circumference, prevalence, trend analysis, NHANES

## Abstract

**Introduction:**

Although waist-to-height ratio (WHtR) has established association with cardiometabolic disease, the trend of changes in elevated WHtR among general population have not been examined adequately.

**Methods:**

This study examined the prevalence of elevated WHtR and waist circumference (WC) and their trends over time using Joinpoint regression models among adults who participated in the United States National Health and Nutrition Examination Survey (U.S. NHANES) 1999–2018. We performed weighted logistic regression to identify the association between central obesity subtypes and the prevalence of comorbidities, including diabetes, chronic kidney disease, hypertension, cardiovascular disease, and cancer.

**Results:**

The prevalence of elevated WHtR has increased from 74.8% in 1999–2000 to 82.7% in 2017–2018 while elevated WC also increased from 46.9% in 1999–2000 to 60.3% in 2017–2018. Men, older adults, former smokers, and people with lower education levels were more likely to have elevated WHtR. A total of 25.5% of American adults had normal WC but elevated WHtR, and they had a significantly higher chance of suffering from diabetes (odds ratio [OR] = 2.06 [1.66, 2.55]), hypertension (OR = 1.75 [1.58, 1.93]) and CVD (OR = 1.32 [1.11, 1.57]).

**Discussion:**

In conclusion, the burden of elevated WHtR and WC have been increasing among U.S. adults throughout the years, and the changes have been more significant across most subgroups. It is also notable that approximately a quarter of the population had normal WC but elevated WHtR, which had increased likelihood of having cardiometabolic diseases, especially diabetes. Future clinical practices should pay more attention to this subgroup of the population with overlooked health risks.

## 1. Introduction

Valid and easy-to-measure anthropometric indicators are needed to screen for people with elevated risk of non-communicable diseases, including diabetes, cardiovascular diseases (CVD) and cancer, which confer a significant global health burden (1, 2). Although body mass index (BMI) is a simple and widely used anthropometric index, accumulating evidence suggests that obesity indices such as waist-to-height ratio (WHtR) and waist circumference (WC) have better screening power for cardiometabolic risk (3). WHtR is a better tool than BMI in assessing central obesity and can be better than WC in accounting for the variations in height (4). Receiver operating characteristic curves of two meta-analyses showed that WHtR had better power than BMI and WC in classifying CVD risk factors among adults and children (3, 5). A cross-sectional analysis of baseline data from a Chinese prospective cohort also suggested WHtR to be the best indicator for dyslipidemia and hyperglycemia (6). Another cross-sectional analysis in 21,109 participants using the United States (U.S.) National Health and Nutrition Examination Survey (NHANES) 1999–2014 also suggested WHtR had a better discriminatory power in predicting diabetes than BMI (area under the curve: 0.709 versus 0.654), and a stronger association with the prevalence of diabetes than BMI (7).

Moreover, increasing prospective studies have demonstrated the predictive power of obesity indices on long-term risk of cardiometabolic diseases. For example, a higher WC has been associated with higher all-cause and CVD mortalities regardless of BMI categories (8, 9). Results from the Nurses’ Health Study also supported that elevated WC was positively associated with all-cause, CVD, and cancer mortalities independently of BMI (10). Among 26,607 participants from the Alberta’s Tomorrow Project cohort, the association between BMI and all-cancer risk became insignificant after adjusting for WC, especially among women (11). In terms of WHtR, it may be associated with a higher risk of diabetes (12), CVD morbidity (13) and liver cancer in prospective cohorts (14). Another cohort analysis among 109,536 postmenopausal women participated in the Women’s Health Initiative had shown a higher risk for CVD events when WHtR was ≥0.5 (Hazard ratio 1.29, 95% confidence interval [CI] 1.22 to 1.36), and the magnitude of association was comparable to WC (HR 1.23, 95% CI 1.17 to 1.30) and possibly stronger than BMI-classified obesity (HR 1.19, 95% CI 1.11 to 1.27) (15).

Although growing studies have indicated the utility of WHtR in early screening for individuals with elevated health risks, several research questions still have not been answered. First, previous studies have examined the changes in WHtR over time among Australian children (15, 16), Chinese women of childbearing age (17), and Chinese adults (18), but the temporal analyses using the U.S. NHANES data did not include WHtR (19, 20). Studies on the prevalence of elevated WHtR in general population in Western countries are inadequate. The second research question is the prevalence of people with normal WC but having elevated WHtR in the general population, as well as their associations with comorbidities. Several studies have investigated the association between normal weight central obesity (people with normal BMI but having excessive abdominal fat), cardiometabolic health and cause-specific mortality (21–23). The key missing information in these studies was the proportion of individuals with central obesity classified by WHtR, but they were defined as normal based on their WC. If this group of individuals also have elevated risk of cardiometabolic diseases, such as higher risk of diabetes and CVD, WHtR should be routinely screened.

To address these aforementioned knowledge gaps, we have conducted the present study to examine the changes in the prevalence of elevated WHtR and WC among U.S. adults using a nationally representative sample. We also demonstrated the prevalence of people with normal WC but having elevated WHtR, and whether this subgroup of population may have significantly higher risk of comorbidities.

## 2. Materials and methods

### 2.1. Study population

NHANES is a nationally representative cross-sectional survey designed to monitor the health and nutritional status of the resident civilian noninstitutionalized U.S. population of the entire nation. The formulation and review of the NHANES program complies with the U.S. Department of Health and Human services’ policy to protect human research subjects (45 CFR 46, available from https://www.hhs.gov/ohrp/regulations-and-policy/regulations/45-cfr-46/index.html, accessed on 10 June 2021). The National Center for Health Statistics Research Ethics Review Board (NCHS ERB) reviewed and approved the study (NCHS ERB protocol number #2005–06 and #2011–17, available from https://www.cdc.gov/nchs/nhanes/irba98.htm, accessed on 10 June 2021). Informed consent was obtained from participants upon recruitment.

We included adults (aged ≥20) from NHANES cycles 1999 to 2018 in this study. Pregnant women and participants without data on WHtR or WC were excluded. To perform the logistic regression, we further excluded those with missing covariate data, including demographic variables and comorbidities. This study was reported as per the Strengthening the Reporting of Observational Studies in Epidemiology (STROBE) guideline for cross-sectional study.

### 2.2. Anthropometric assessment

The anthropometric indexes included WHtR and WC. Anthropometric measurements were performed at the Mobile Examination Center using standardized anthropometry examination procedures and calibrated equipment (24). A fixed stadiometer with a vertical backboard and a moveable headboard was used for height measurement. Height measurements were taken using different equipment during different time periods. Between 1999 and 2006, a Seca electronic stadiometer was used, while starting in 2007, a ProScale Inductive Incremental scale with a ProScale digital measurement device connected to the acrylic headpiece was utilized. Although the equipment changed, the measurement discrepancies were minor and negligible. WC was measured at minimal respiration by positioning a measuring tape in a horizontal plane at the level of right above the uppermost lateral border of the right ilium. Between 1999 and 2006, the measurements were carried out by a single examiner who relied on a wall-mounted mirror to ensure accurate horizontal alignment of the measuring tape. However, starting from 2007, the measurement procedure was revised to include an additional step. In addition to the use of the mirror, the recorder was now also required to move to the participants’ left side to verify the correct placement of the measuring tape. This amendment to the Anthropometry Procedures Manual was intended to improve accuracy and reduce measurement error, rather than to substantially change the measurement itself. As a result, the impact of these changes on data analysis is typically minor and can be neglected. WHtR was defined as the waist circumference divided by the height, both in the same units. WC and WHtR were dichotomized by the common cut-off points among adult population. The conventional indicators for central obesity included elevated WHtR (WHtR≥0.5) (25), and elevated WC (≥102 cm for men and ≥ 88 cm for women) (26).

### 2.3. Sociodemographic and lifestyle characteristics

Questionnaires were administered by trained interviewers to collect sociodemographic information and medical history. These included age (20–39, 40–59, ≥60 years), sex (men or women), race/ethnicity (Non-Hispanic White, Non-Hispanic Black, other Hispanic or other race), educational attainment (less than high school, high school, at least some college), poverty-income-ratio (PIR, dichotomized as <1 or ≥ 1), smoking status (categorized as never, former, or current smoker). For adults 20 and older, smoking status were asked by trained interviewers using a Computer-Assisted Personal Interviewing (CAPI) system in the home. In this study, smoking status grouped as never smoker (<100 cigarettes in the entire life), former smoker (≥100 cigarettes and non-smoker currently), and current smoker (≥100 cigarettes and smoking currently) (27). PIR, a socioeconomic status index, is the ratio of the family’s self-reported income to the family’s appropriate poverty threshold according to the U.S. Census Bureau, and PIR values of 1.00 or greater indicating people above the poverty threshold (28).

### 2.4. Definition of pre-existing comorbidities

Diabetes was defined as the presence of at least one of following criteria: (1) fasting plasma glucose ≥7.0 mmol/L (126 mg/dL); (2) hemoglobin A1c (HbA1c) ≥6.5% (48 mmol/mol); (3) oral glucose tolerance test ≥11.1 mmol/L (200 mg/dL); (4) for a patient with classic symptoms of hyperglycemia or hyperglycemic crisis, having a random plasma glucose ≥11.1 mmol/L (200 mg/dL) (29). Chronic kidney disease (CKD) was defined as eGFR <60 mL/min per 1.73 m2 or a urinary albumin-to-creatinine ratio > 30 mg/g (30). Hypertension was defined as the presence of at least one of the following conditions: (1) systolic blood pressure ≥ 140 mmHg or diastolic blood pressure ≥ 90 mmHg; (2) current use of medication to treat hypertension; and/or (3) self-reported hypertension (31). Any CVD was considered to be present if the participant self-reported prior coronary heart disease, heart failure, or stroke as informed by a doctor (32). Cancer was defined as self-reported history of cancer or malignancy.

### 2.5. Statistical analyses

Descriptive statistics were used to present the frequency and proportion of the sociodemographic factors. We calculated the weighted prevalence of elevated WHtR and WC using the sample weights created for the NHANES study. The weights account for the complex survey design (including oversampling), survey nonresponse, and post-stratification in order to ensure that calculated estimates are representative of the U.S. civilian noninstitutionalized population. We used Joinpoint regression models and examined changes in the prevalence of elevated WHtR and elevated WC over time expressed as average annual percentage change (AAPC) for the entire study period and annual percentage change (APC) for each linear trend segment detected (33, 34). The minimum number of observations from a Joinpoint to either end of the data (excluding the first or last Joinpoint if it falls on an observation) and between two Joinpoints (excluding any Joinpoint if it falls on an observation) was set as 2. Tests of coincidence were performed in pairwise comparison to see whether the changing trend of WHtR and WC among various subgroups were statistically significant, *p* value <0.05 (35).

We conducted a series of subgroup analyses on estimating trends in prevalence of elevated WHtR and WC over time, including the socio-demographics (sex, ethnicity, age, education level, PIR), lifestyle characteristics (smoking status), and with or without established-comorbidities (diabetes, CKD, hypertension, CVD, and cancer). We further defined four subtypes of central obesity by elevated WHtR and elevated WC and examined changes in prevalence of each subtype of central obesity over time (normal WC and normal WHtR, normal WC and elevated WHtR, elevated WC and normal WHtR, elevated WC and elevated WHtR).

We additionally performed logistic regression to identify the association of central obesity subtypes and prevalence of comorbidities. Logistic regression models were adjusted for socio-demographics (sex, ethnicity, age, education level, PIR), lifestyle characteristics (smoking status), and other included comorbidities. Two-sided *p* values <0.05 were considered statistically significant. All statistical analyses were completed using R version 4.0.3 software (R Foundation for Statistical Computing, Vienna, Austria) and the Joinpoint Regression Program, Version 4.9.1.0 -April 2022; Statistical Methodology and Applications Branch, Surveillance Research Program, National Cancer Institute (35).

## 3. Results

### 3.1. Characteristics of participants

In the analysis on trends in prevalence of elevated WHtR and elevated WC, among 101,316 participants in the NHANES survey from 1999 to 2018, those age < 20 years (*n* = 46,235), pregnant women (*n* = 1,442), and participants without data on WHtR or WC (*n* = 5,790) were excluded. A total of 47,849 participants were included in the trend analysis (Supplementary Figure S1). Over 20 years, the mean (SD) age ranged from 48.4 (17.4) and 51.2 (17.5) years, while the percentage of men varied between 48.7 and 51.3%. The prevalence of comorbidities varied in 11.8 to 21.1% for diabetes, 10.4 to 14.2% for CKD, 39.9 to 46.7% for hypertension, 9.5 to 13.1% for CVD, and 7.9 to 10.1% for cancer (Table 1). To perform the association analyses of subtypes of central obesity with the prevalence of comorbidities, 43,294 participants without missing covariate data were included, with characteristics presented in Supplementary Table S1.

**Table 1:** Characteristics of participants in NHANES 1999–2018.

	1999–2000	2001–2002	2003–2004	2005–2006	2007–2008	2009–2010	2011–2012	2013–2014	2015–2016	2017–2018	Overall
	4022	4378	4254	4186	5317	5632	4916	5206	5050	4888	47849
Men, *n* (%)	2005 (49.9)	2199 (50.2)	2153 (50.6)	2148 (51.3)	2635 (49.6)	2764 (49.1)	2473 (50.3)	2533 (48.7)	2470 (48.9)	2404 (49.2)	23784 (49.7)
*Ethnicity, n(%)*											
Mexican american	1087 (27.0)	920 (21.0)	847 (19.9)	817 (19.5)	911 (17.1)	1,043 (18.5)	487 (9.9)	700 (13.4)	882 (17.5)	646 (13.2)	8340 (17.4)
Other hispanic	253 (6.3)	179 (4.1)	130 (3.1)	130 (3.1)	596 (11.2)	581 (10.3)	505 (10.3)	471 (9.0)	682 (13.5)	449 (9.2)	3976 (8.3)
Non-Hispanic White	1794 (44.6)	2284 (52.2)	2257 (53.1)	2104 (50.3)	2,93 (46.9)	2724 (48.4)	1805 (36.7)	2230 (42.8)	1674 (33.1)	1696 (34.7)	21061 (44.0)
Non-Hispanic Black	764 (19.0)	860 (19.6)	838 (19.7)	969 (23.1)	1101 (20.7)	976 (17.3)	1284 (26.1)	1056 (20.3)	1,050 (20.8)	1,150 (23.5)	10,048 (21.0)
Other race	124 (3.1)	135 (3.1)	182 (4.3)	166 (4.0)	216 (4.1)	308 (5.5)	835 (17.0)	749 (14.4)	749 (14.4)	947 (19.4)	4424 (9.2)
*Age group, (years)*	50.9 (18.4)	49.4 (18.2)	51.0 (19.1)	49.2 (18.4)	50.4 (17.6)	49.4 (17.6)	48.4 (17.4)	48.8 (17.2)	49.3 (17.4)	51.2 (17.5)	49.8 (17.9)
20–39	1282 (31.9)	1477 (33.7)	1380 (32.4)	1454 (34.7)	1706 (32.1)	1864 (33.1)	1744 (35.5)	1761 (33.8)	1705 (33.8)	1466 (30.0)	15839 (33.1)
40–59	1224 (30.4)	1491 (34.1)	1275 (30.0)	1385 (33.1)	1739 (32.7)	1937 (34.4)	1,659 (33.7)	1834 (35.2)	1709 (33.8)	1577 (32.3)	15830 (33.1)
≥60	1516 (37.7)	1410 (32.2)	1599 (37.6)	1347 (32.2)	1872 (35.2)	1831 (32.5)	1513 (30.8)	1611 (30.9)	1,636 (32.4)	1845 (37.7)	16180 (33.8)
*Smoking status, n (%)*											
Never smoker	2080 (51.7)	2192 (50.1)	2098 (49.3)	2,159 (51.6)	2773 (52.2)	3028 (53.8)	2794 (56.8)	2930 (56.3)	2918 (57.8)	2,817 (57.6)	25,789 (53.9)
Former smoker	1082 (26.9)	1152 (26.3)	1157 (27.2)	1068 (25.5)	1322 (24.9)	1376 (24.4)	1110 (22.6)	1202 (23.1)	1158 (22.9)	1,170 (23.9)	11,797 (24.7)
Current smoker	853 (21.2)	1028 (23.5)	997 (23.4)	957 (22.9)	1216 (22.9)	1228 (21.8)	1005 (20.4)	1073 (20.6)	966 (19.1)	901 (18.4)	10,224 (21.4)
*Education level, n (%)*											
Less than high school	1,583 (39.4)	1307 (29.9)	1241 (29.2)	1133 (27.1)	1643 (30.9)	1598 (28.4)	1138 (23.1)	1105 (21.2)	1174 (23.2)	957 (19.6)	12,879 (26.9)
High school graduate	915 (22.7)	1,032 (23.6)	1,076 (25.3)	1009 (24.1)	1312 (24.7)	1298 (23.0)	1032 (21.0)	1160 (22.3)	1097 (21.7)	1,172 (24.0)	11,103 (23.2)
College or above	1,524 (37.9)	2038 (46.6)	1937 (45.5)	2044 (48.8)	2362 (44.4)	2736 (48.6)	2746 (55.9)	2941 (56.5)	2779 (55.0)	2,759 (56.4)	23,866 (49.8)
*Poverty income ratio, n (%)*											
In poverty (< 1.00)	694 (17.3)	674 (15.4)	722 (17.0)	662 (15.8)	1001 (18.8)	1123 (19.9)	1129 (23.0)	1062 (20.4)	993 (19.7)	773 (15.8)	8,833 (18.5)
Not in poverty (≥ 1.00)	2761 (68.6)	3396 (77.6)	3286 (77.2)	3339 (79.8)	3847 (72.4)	3983 (69.0)	3393 (69.0)	3750 (72.0)	3566 (70.6)	3501 (71.6)	34822 (72.8)
*Comorbidities, n (%)*											
Diabetes, *n* (%)	501 (12.5)	517 (11.8)	591 (13.9)	619 (14.8)	987 (18.6)	993 (17.6)	898 (18.3)	910 (17.5)	1007 (19.9)	1030 (21.1)	8053 (16.8)
Chronic kidney disease, *n* (%)	570 (14.2)	523 (11.9)	503 (11.8)	521 (12.4)	726 (13.7)	584 (10.4)	596 (12.1)	599 (11.5)	604 (12.0)	649 (13.3)	5875 (12.3)
Hypertension, n (%)	1703 (42.3)	1745 (39.9)	1882 (44.2)	1690 (40.4)	2294 (43.1)	2295 (40.7)	2036 (41.4)	2192 (42.1)	2139 (42.4)	2281 (46.7)	20257 (42.3)
Cardiovascular disease, *n* (%)	446 (11.1)	448 (10.2)	557 (13.1)	461 (11.0)	597 (11.2)	577 (10.2)	467 (9.5)	502 (9.6)	516 (10.2)	578 (11.8)	5,149 (10.8)
Cancer, *n* (%)	318 (7.9)	405 (9.3)	401 (9.4)	357 (8.5)	514 (9.7)	562 (10.0)	402 (8.2)	492 (9.5)	479 (9.5)	494 (10.1)	4424 (9.2)

### 3.2. Trends in prevalence of elevated WHtR and WC

**Figure 1:**
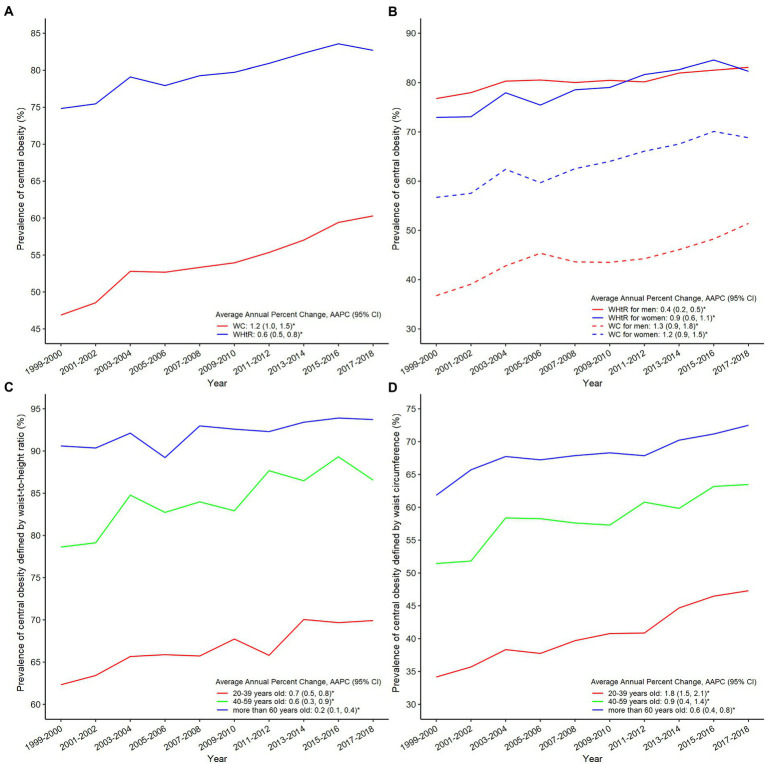
Trends of prevalence of elevated waist-to-height ratio and waist circumference in NHANES 1999–2018 in overall population **(A)** and when stratified by sex **(B)**; prevalence of elevated WHtR **(C)** and WC **(D)** stratified by age.

The overall standardized prevalence increased from 74.8 to 82.7% (AAPC 0.6, 95% CI 0.5 to 0.8) for elevated WHtR and from 46.9 to 60.3% (AAPC 1.2, 95% CI 1.0 to 1.5) for elevated WC during 1999–2018 (Figure 1A; Supplementary Table S2). Similar increased trends in elevated WHtR and elevated WC were observed in both sexes, with the significantly more rapid increment of elevated WHtR in women (AAPC 0.9, 95% CI 0.6 to 1.1 versus AAPC 0.4, 95% CI 0.2 to 0.5 in men, *p* = 0.010) and elevated WC in men (AAPC 1.3, 95% CI 0.9 to 1.8 versus AAPC 1.2, 95% CI 0.9 to 1.5 in women, *p* < 0.001) (Figure 1B). Compared with women, men had the significantly higher prevalence of elevated WHtR (83.1% versus 72.9%, p < 0.001) (Supplementary Table S2) but lower prevalence of elevated WC (51.4% versus 68.8%, p < 0.001) in 2017–2018 (Supplementary Table S3). Similar increasing trends were observed in all age-groups (Figures 1C,D). Compared with the youngest participants (20–39 years), those aged ≥60 years had the highest prevalence of elevated WHtR (93.7% versus 69.9%) (Supplementary Table S2) and elevated WC (72.5% versus 47.3%) (Supplementary Table S3) in 2017–2018, while the youngest participants (aged 20–39) had the most rapid increment of elevated WC (AAPC 1.8, 95% CI 1.5 to 2.1, Figure 1D).

### 3.3. Subgroups analyses of trends in elevated WHtR and WC

**Figure 2:**
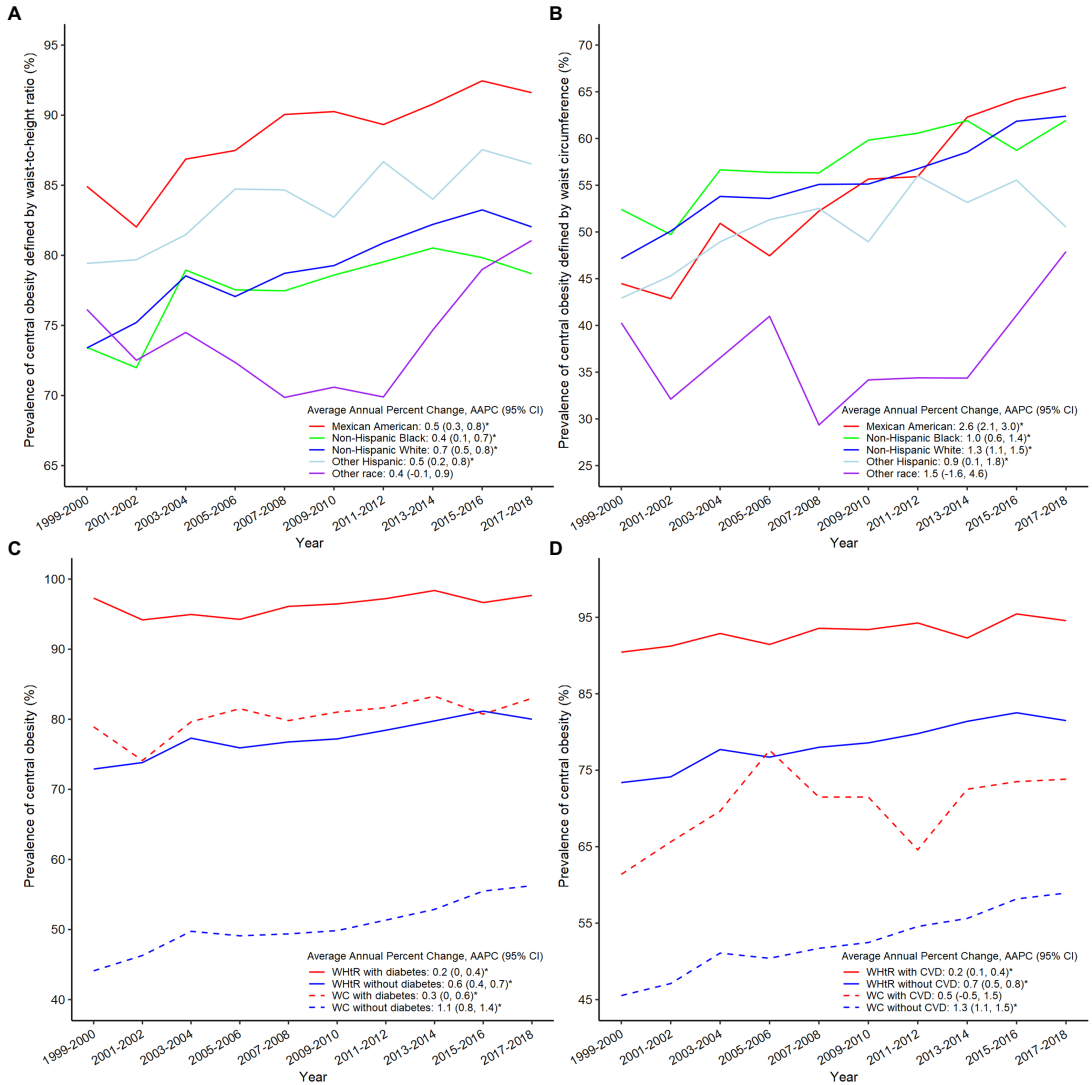
Trends of prevalence of elevated WHtR **(A)** and WC **(B)** in NHANES 1999–2018 stratified by ethnicity; prevalence of elevated WHtR and WC stratified by the status of diabetes **(C)** or CVD **(D)**.

There were significant variations in trends of elevated WHtR (Figure 2A; Supplementary Table S2) and elevated WC (Figure 2B; Supplementary Table S3) by ethnicity and comorbidities. Mexican American had the highest prevalence of elevated WHtR (91.6%) and elevated WC (65.5%) in 2017–2018 (Figures 2A,B; Supplementary Tables S2, S3). Compared with and without established comorbidities, those with established comorbidities had the highest prevalence of elevated WHtR and elevated WC (97.7% versus 80.0 and 83.0% versus 56.3% for diabetes, and 94.6% versus 81.5 and 73.8% versus 58.9% for CVD, Figures 2C,D and Supplementary Tables S2-S4). The similar patterns of elevated WHtR and elevated WC were also observed by smoke status education, PIR, and other comorbidities such as hypertension, CKD, and cancer (Supplementary Figures S2−S7; Supplementary Tables S2−S4).

### 3.4. Trends in prevalence of central obesity subtypes and their associations with comorbidities

**Figure 3:**
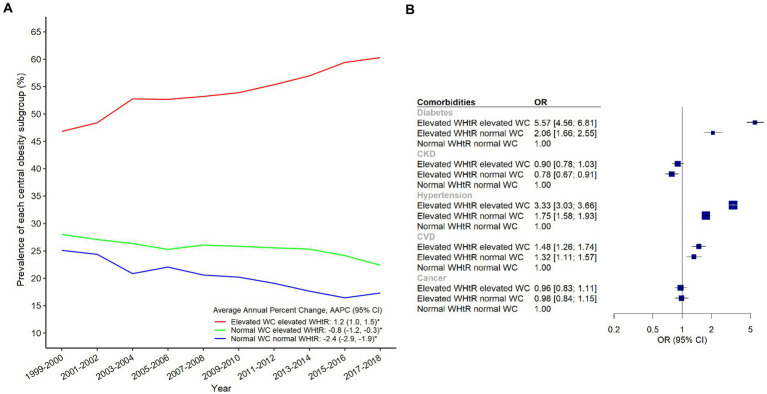
Trends of prevalence of each central obesity subgroup in NHANES 1999–2018 **(A)** and the forest plot of the associations between central obesity subgroups and comorbidities **(B)**.

From 1999 to 2018, the prevalence of normal WC and normal WHtR decreased from 25.1 to 17.3% (AAPC -2.4, 95% CI -2.9 to -1.9), while the prevalence of elevated WHtR and elevated WC increased from 46.8 to 60.3% (AAPC 1.2, 95% CI 1.0 to 1.5). The prevalence of elevated WHtR and normal WC decreased from 28.0 to 22.4% (AAPC -0.8, 95% CI -1.2 to -0.3, Figure 3A and Supplementary Table S5). Compared with participants with normal WHtR and normal WC, those with elevated WHtR and normal WC had higher odds of diabetes (OR 2.06, 95% CI 1.66 to 2.55), hypertension (OR 1.75, 95% CI 1.58 to 1.93) and CVD (OR 1.32, 95% CI 1.11 to 1.57) after adjusting for potential risk factors (Figure 3B). The highest odds of these comorbidities were observed in participants with elevated WHtR and elevated WC. Effect estimates were not shown for adults with normal WHtR and elevated WC due to small sample size (n = 15).

## 4. Discussion

In 1999–2018 among the American adults, the prevalence of central obesity (elevated WHtR and elevated WC) increased across various subgroups by socio-demographics, lifestyle, and comorbidity status. An estimated 22 to 28% of adults had normal WC but elevated WHtR, and these individuals had 1.3 to 2.1 times higher odds of diabetes, hypertension, and CVD. Men, older adults, former smokers, and people with lower education levels were more likely to have elevated WHtR and/or WC.

The variation in central obesity prevalence by sex, age, smoking status and socioeconomic status were in generally consistent with the relationship suggested from previous literature. The increased rate of elevated WHtR and WC among women can be explained by the levels of physical activity and the role of sex hormones on adipose tissue lipolysis (36). Moreover, the dysfunction of adipose tissue and accumulated inflammation related with aging, may explain the linkage between older age and a higher likelihood of central obesity (37). For the relationship between smoking status and obesity, previous literature suggested that the increase in resting metabolic rate due to acute effect of nicotine (38), which may explain the lower rate of elevated WHtR and WC among current smokers. Along with the reduction of nicotine intake, it is likely that individuals resumed normal appetite and increased energy intake after quitting smoking, leading to temporary weight gain (39). However, the present study only examined the cross-sectional variation between smoking status and the rate of central obesity, therefore how the duration of smoking cessation might impact obesity has not been evaluated. For the ethnic differences in obesity rate, disparities between Mexican Americans and non-Hispanic white Americans have also been observed in the systematic review of the trend of BMI-classified obesity in NHANES population (40), which may be related to the weight misperception and lower intention to try weight loss (41, 42). In the same review, it is also found a short leveling-off of BMI-classified obesity between 2009 and 2012 (40), which also agrees with our present analysis using WHtR as adiposity indicator. However, the reason behind warrants further investigation. For the variation of central obesity by education levels, the reasons behind can be limited health literacy, fewer socioeconomic resources to purchase healthier dietary options, and fewer social support (43). Although the observed associations may not be fully consistent with previous literature, the present study has indicated the at-risk population for more efforts on obesity prevention and management. Furthermore, based on the findings from Joinpoint regression model, the prevalence of elevated WHtR and WC increased consistent across subgroups, and the disparities did not attenuate with time. This is also another alarming sign in controlling the obesity pandemic.

Another major finding of the present study is to quantify the proportion of individuals with central obesity but being masked by having normal WC, which may reveal a subgroup of population with overlooked risk for cardiometabolic diseases. From the logistic regression, people with diabetes, hypertension and CVD were positively associated with the odds of having elevated WHtR and WC, which was consistent with previous meta-analysis and NHANES analysis (3, 7), suggesting that WHtR may be a better screening tool than WC in identifying participants with diabetes risk. Although WC is an established indicator for assessing central obesity (≥ 102 cm for men and ≥ 88 cm for women), it has not accounted for the variation in body height. For people with different heights, the proportion of abdominal fat reflected by waist circumference may differ. In other words, the use of WC in screening may miss out individuals with WHtR≥0.5 but having smaller body size, i.e., men with height lower than 204 cm and women with height lower than 176 cm. From the logistic regression, people with elevated WHtR but normal WC were more likely to suffer from diabetes, hypertension, and CVD, especially for diabetes, despite having normal WC. This observation has provided evidence to support that WHtR may be a better screening tool than WC in identifying participants with diabetes, hypertension, and CVD risk. Apart from the surveillance of elevated WHtR in national samples, incorporating WHtR into routine clinical screening, and potentially the risk algorithm of calculating cardiometabolic risk becomes necessary. WHtR can be easily derived from the data of BMI and WC and is convenient to measure and interpret (5). This great advantage in practice allows quick identification of individuals with cardio-metabolic risk.

The main strength of this study is the use of nationally representative NHANES data, which provides an estimate of national prevalence in elevated WHtR and WC, as well as the high-risk population being masked by having normal WC. The NHANES anthropometry measurement data analyzed in this study was of exceptional quality. To maintain accuracy and comparability over time, NHANES has established standardized examination procedures for measuring height and waist circumference. These procedures entail specific techniques for taking measurements and necessitate equipment calibration. While equipment and techniques for measuring height and waist circumference have undergone minor improvements, the objective has been to enhance accuracy and reduce measurement error, as outlined in the Methods section. This study has several limitations. First, anthropometric data were only collected at single time point and the individual trajectory of adiposity indicators were not examined. Second, we were not able to establish a causal relationship between WHtR, WC and long-term risk of chronic disease due to the repeated cross-sectional design. Third, the history of comorbidities was self-reported, and the prevalence of comorbidities may be underestimated in this study.

## 5. Conclusion

The overall prevalence of elevated WHtR (82.7%) and WC (60.3%) was high among U.S. adults, and there was a significant increasing trend in 1999–2018, with consistent patterns by socio-demographics, lifestyle, and comorbidity status. Women, older adults, former smokers, people with lower education levels and comorbidities were more likely to have elevated WHtR. The present study also indicated that people with elevated WHtR but with normal WC had a higher likelihood of having diabetes, hypertension, and CVD.

## Data availability statement

Publicly available datasets were analyzed in this study. This data can be found at: https://wwwn.cdc.gov/nchs/nhanes/Default.aspx.

## Author contributions

AY and KL designed the research and revised the manuscript critically. BY and JY conducted statistical analysis and led the writing of the manuscript. BY, JY, MW, JR, VC, MK, and KL drafted the manuscript. All authors contributed to the interpretation of the results and critical revision of the manuscript for important intellectual content and approved the final version of the manuscript.

## Funding

KL was supported by Funding for Research Institutes (grant number: CD69), Undergraduate Research and Innovation Scheme (grant number: TAAU) and Start-up Fund for RAPs under the Strategic Hiring Scheme (grant number: BD8H). The funders had no role in study design, data collection and analysis, decision to publish, or preparation of the manuscript.

## Conflict of interest

The authors declare that the research was conducted in the absence of any commercial or financial relationships that could be construed as a potential conflict of interest.

## Publisher’s note

All claims expressed in this article are solely those of the authors and do not necessarily represent those of their affiliated organizations, or those of the publisher, the editors and the reviewers. Any product that may be evaluated in this article, or claim that may be made by its manufacturer, is not guaranteed or endorsed by the publisher.
