# Supplementary material for: Trends in elevated waist-to-height ratio and waist circumference in U.S. adults and their associations with cardiometabolic diseases and cancer, 1999–2018
Source: Front Nutr. 2023 Apr 11;10:1124468. doi: 10.3389/fnut.2023.1124468 (PMC10126508; doi:10.3389/fnut.2023.1124468)
Supplement: Supplementary file 1 [file Data_Sheet_1.docx]

Supplementary Material

Trends in elevated waist-to-height ratio and waist circumference in U.S. Adults and their associations with comorbidities, 1999–2018

Bo Yang, MSc^1#^, Jingli Yang, BSc^2#^, Martin Ming-him Wong, BSc^3^, Juwel Rana, MPH^4,5^, Qinghua Yang, MD^6^, Aimin Yang, PhD^7*^, Kenneth Lo, PhD^8,9*^

*** Correspondence:**
Aimin Yang, Ph.D., Department of Medicine and Therapeutics, The Chinese University of Hong Kong, Prince of Wales Hospital, Hong Kong SAR, China
[aiminyang@cuhk.edu.hk](mailto:aiminyang@cuhk.edu.hk), +852 9240-2640
Kenneth Lo, Ph.D., Department of Food Science and Nutrition, The Hong Kong Polytechnic University, Hong Kong SAR, China
[kenneth.kh.lo@polyu.edu.hk](mailto:kenneth.kh.lo@polyu.edu.hk), +852 3400-8778

NHANES 1999-2018

*n* = 101,316

Exclude: *n* = 46,235

- participants aged <20 years

Adults aged 20 years or older

*n* = 55,081

Exclude: *n* = 1,442

- pregnant women

Adults excluding pregnant women

*n* = 53,639

Adults in trend analysis

*n* = 47,849

Exclude: *n* = 4,555, with missing:

- smoking status (*n* = 39)
- education level (*n* = 1)
- poverty income ratio (*n* = 4,194)
- history of comorbidities:

- cardiovascular disease (*n* = 6)

- chronic kidney disease (*n* = 326)

- hypertension (*n* = 13)

- cancer (*n* = 45)

Adults with complete covariate data for association analyses

*n* = 43,294

**Figure S1.** Selection of study participants^[[1]](#footnote-1)^


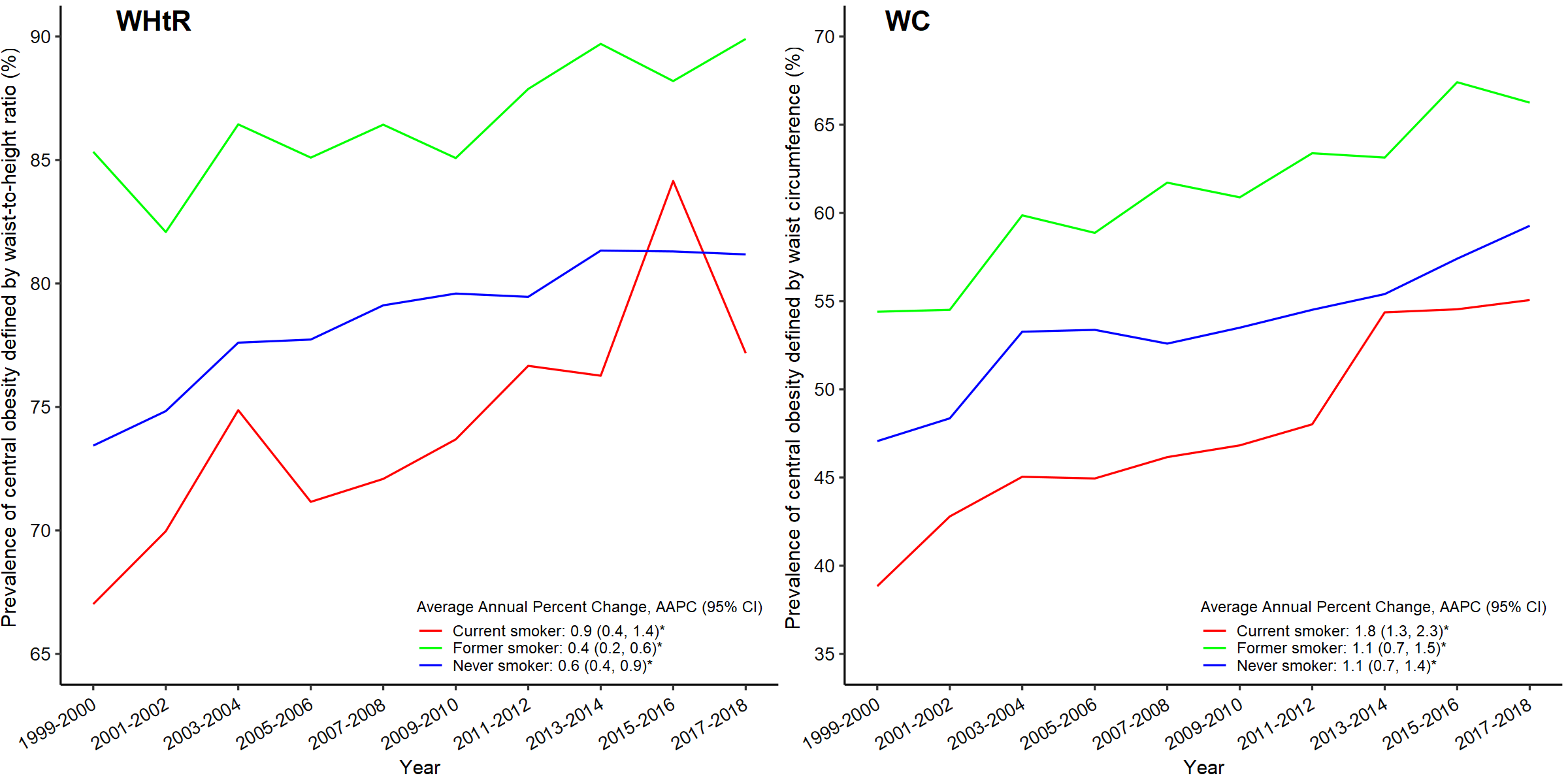


**Figure S2.** Prevalence of elevated waist circumference and waist-to-height ratio stratified by smoking status in American adults, 1999-2018


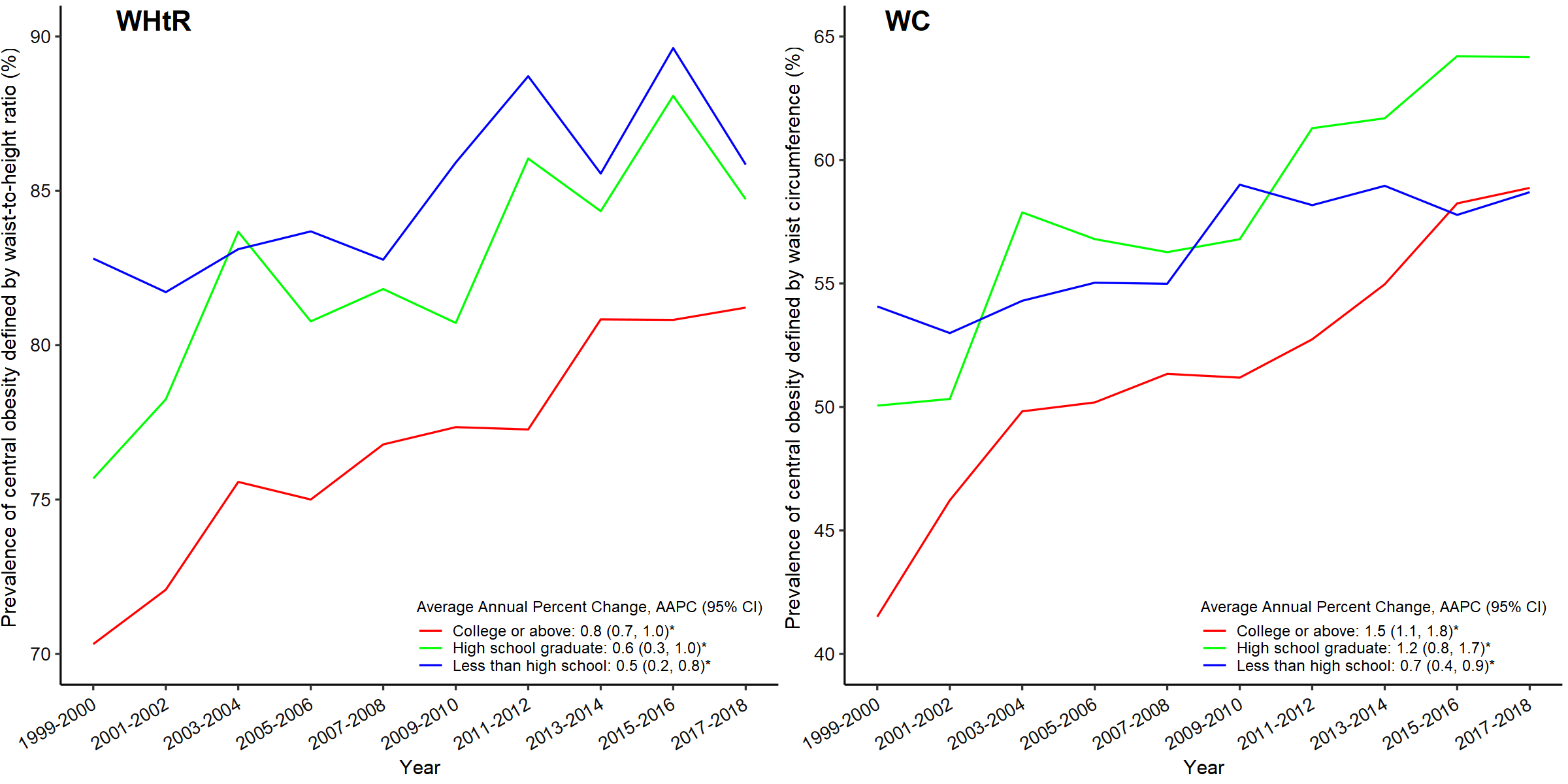


**Figure S3.** Prevalence of elevated waist circumference and waist-to-height ratio stratified by education level in American adults, 1999-2018


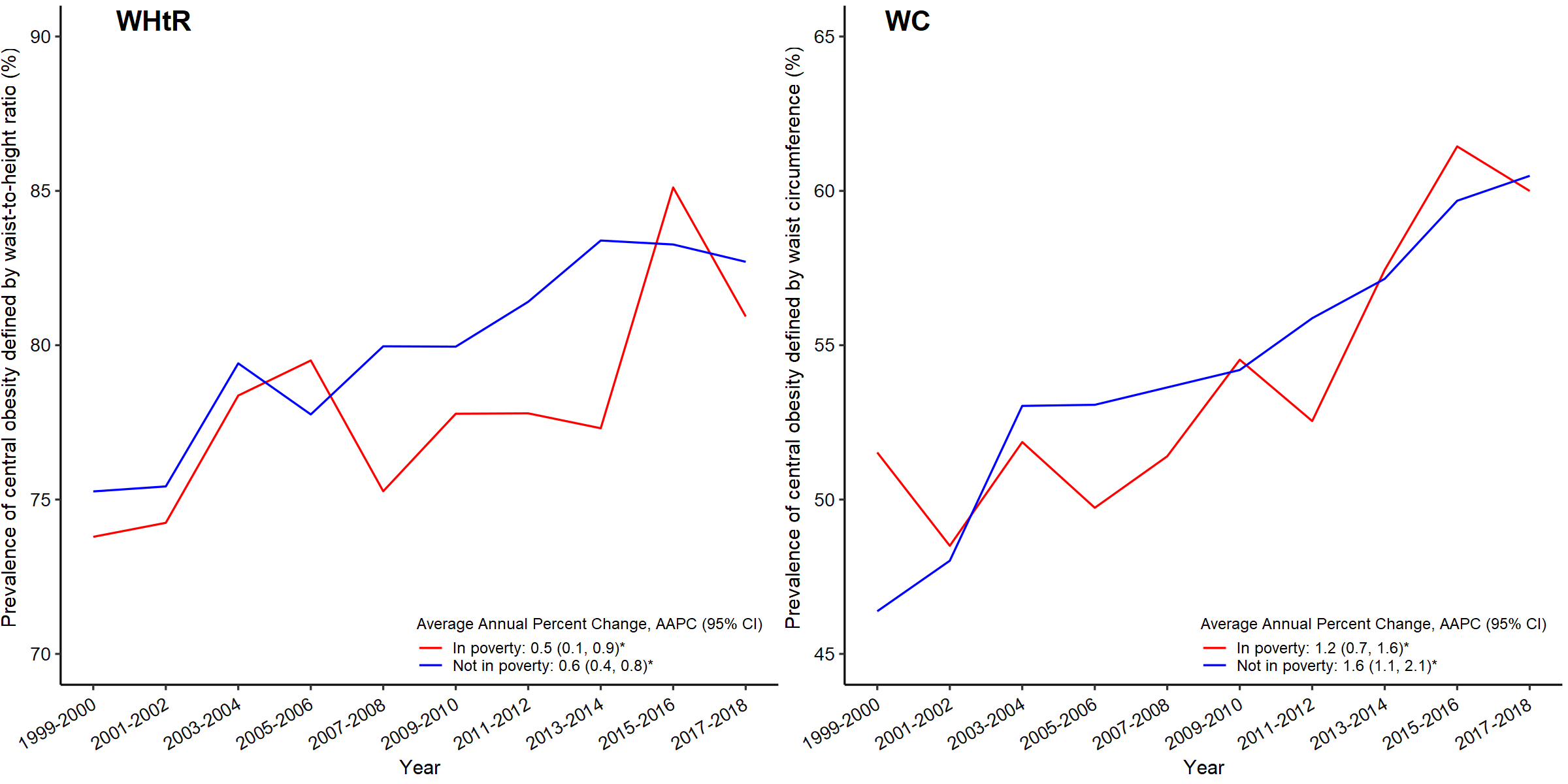


**Figure S4.** Prevalence of elevated waist circumference and waist-to-height ratio stratified by poverty income ratio in American adults, 1999-2018

**Table S1.** Characteristics of participants included in the logistic regression

|  | 1999-2000 | 2001-2002 | 2003-2004 | 2005-2006 | 2007-2008 | 2009-2010 | 2011-2012 | 2013-2014 | 2015-2016 | 2017-2018 | Overall |
| --- | --- | --- | --- | --- | --- | --- | --- | --- | --- | --- | --- |
|  | 3403 | 4016 | 3928 | 3977 | 4810 | 5085 | 4491 | 4797 | 4530 | 4257 | 43294 |
| Men, n (%) | 1705 (50.1) | 2037 (50.7) | 1993 (50.7) | 2039 (51.3) | 2389 (49.7) | 2501 (49.2) | 2252 (50.1) | 2335 (48.7) | 2223 (49.1) | 2096 (49.2) | 21570 (49.8) |
| Ethnicity, n (%) |  |  |  |  |  |  |  |  |  |  |  |
| Mexican American | 901 (26.5) | 840 (20.9) | 800 (20.4) | 755 (19.0) | 808 (16.8) | 892 (17.5) | 438 (9.8) | 606 (12.6) | 770 (17.0) | 533 (12.5) | 7343 (17.0) |
| Other Hispanic | 213 (6.3) | 161 (4.0) | 116 (3.0) | 125 (3.1) | 506 (10.5) | 478 (9.4) | 456 (10.2) | 417 (8.7) | 597 (13.2) | 368 (8.6) | 3437 (7.9) |
| Non-Hispanic White | 1562 (45.9) | 2130 (53.0) | 2087 (53.1) | 2006 (50.4) | 2306 (47.9) | 2569 (50.5) | 1709 (38.1) | 2113 (44.0) | 1563 (34.5) | 1564 (36.7) | 19609 (45.3) |
| Non-Hispanic Black | 623 (18.3) | 764 (19.0) | 767 (19.5) | 930 (23.4) | 994 (20.7) | 874 (17.2) | 1144 (25.5) | 972 (20.3) | 922 (20.4) | 963 (22.6) | 8953 (20.7) |
| Other race | 104 (3.1) | 121 (3.0) | 158 (4.0) | 161 (4.0) | 196 (4.1) | 272 (5.3) | 744 (16.6) | 689 (14.4) | 678 (15.0) | 829 (19.5) | 3952 (9.1) |
| Age group, Mean (SD) | 50.3 (18.3) | 49.1 (18.1) | 51.0 (18.9) | 48.9 (18.2) | 50.3 (17.6) | 49.3 (17.7) | 48.2 (17.4) | 48.8 (17.2) | 48.9 (17.3) | 51.3 (17.4) | 49.6 (17.8) |
| 20-39, n (%) | 1115 (32.8) | 1369 (34.1) | 1268 (32.3) | 1394 (35.1) | 1555 (32.3) | 1696 (33.4) | 1616 (36.0) | 1634 (34.1) | 1555 (34.3) | 1280 (30.1) | 14482 (33.5) |
| 40-59, n (%) | 1061 (31.2) | 1379 (34.3) | 1199 (30.5) | 1325 (33.3) | 1573 (32.7) | 1746 (34.3) | 1516 (33.8) | 1681 (35.0) | 1548 (34.2) | 1378 (32.4) | 14406 (33.3) |
| ≥60, n (%) | 1227 (36.1) | 1268 (31.6) | 1461 (37.2) | 1258 (31.6) | 1682 (35.0) | 1643 (32.3) | 1359 (30.3) | 1482 (30.9) | 1427 (31.5) | 1599 (37.6) | 14406 (33.3) |
| Smoking status, n (%) |  |  |  |  |  |  |  |  |  |  |  |
| Never smoker | 1777 (52.2) | 2003 (49.9) | 1937 (49.3) | 2055 (51.7) | 2506 (52.1) | 2707 (53.2) | 2556 (56.9) | 2675 (55.8) | 2607 (57.5) | 2424 (56.9) | 23247 (53.7) |
| Former smoker | 898 (26.4) | 1067 (26.6) | 1073 (27.3) | 1019 (25.6) | 1204 (25.0) | 1267 (24.9) | 1015 (22.6) | 1129 (23.5) | 1058 (23.4) | 1055 (24.8) | 10785 (24.9) |
| Current smoker | 728 (21.4) | 946 (23.6) | 918 (23.4) | 903 (22.7) | 1100 (22.9) | 1111 (21.8) | 920 (20.5) | 993 (20.7) | 865 (19.1) | 778 (18.3) | 9262 (21.4) |
| Education level, n (%) |  |  |  |  |  |  |  |  |  |  |  |
| Less than high school | 1288 (37.8) | 1172 (29.2) | 1135 (28.9) | 1054 (26.5) | 1459 (30.3) | 1376 (27.1) | 996 (22.2) | 970 (20.2) | 1007 (22.2) | 780 (18.3) | 11237 (26.0) |
| High school graduate | 774 (22.7) | 954 (23.8) | 973 (24.8) | 962 (24.2) | 1178 (24.5) | 1174 (23.1) | 932 (20.8) | 1071 (22.3) | 988 (21.8) | 1032 (24.2) | 10038 (23.2) |
| College or above | 1341 (39.4) | 1890 (47.1) | 1820 (46.3) | 1961 (49.3) | 2173 (45.2) | 2535 (49.9) | 2563 (57.1) | 2756 (57.5) | 2535 (56.0) | 2445 (57.4) | 22019 (50.9) |
| Poverty income ratio, n (%) |  |  |  |  |  |  |  |  |  |  |  |
| In poverty (< 1.00) | 682 (20.0) | 649 (16.2) | 705 (17.9) | 658 (16.5) | 988 (20.5) | 1119 (22.0) | 1118 (24.9) | 1058 (22.1) | 986 (21.8) | 770 (18.1) | 8733 (20.2) |
| Not in poverty (≥ 1.00) | 2721 (80.0) | 3367 (83.8) | 3223 (82.1) | 3319 (83.5) | 3822 (79.5) | 3966 (78.0) | 3373 (75.1) | 3739 (77.9) | 3544 (78.2) | 3487 (81.9) | 34561 (79.8) |
| Diabetes, n (%) | 411 (12.1) | 463 (11.5) | 540 (13.7) | 577 (14.5) | 887 (18.4) | 889 (17.5) | 810 (18.0) | 844 (17.6) | 900 (19.9) | 900 (21.1) | 7221 (16.7) |
| Chronic kidney disease, n (%) | 479 (14.1) | 474 (11.8) | 465 (11.8) | 487 (12.2) | 653 (13.6) | 525 (10.3) | 526 (11.7) | 556 (11.6) | 531 (11.7) | 574 (13.5) | 5270 (12.2) |
| Hypertension, n (%) | 1435 (42.2) | 1589 (39.6) | 1741 (44.3) | 1605 (40.4) | 2089 (43.4) | 2076 (40.8) | 1848 (41.1) | 2018 (42.1) | 1894 (41.8) | 1980 (46.5) | 18275 (42.2) |
| Cardiovascular disease, n (%) | 371 (10.9) | 399 (9.9) | 510 (13.0) | 427 (10.7) | 536 (11.1) | 529 (10.4) | 421 (9.4) | 468 (9.8) | 464 (10.2) | 517 (12.1) | 4642 (10.7) |
| Cancer, n (%) | 270 (7.9) | 368 (9.2) | 367 (9.3) | 334 (8.4) | 467 (9.7) | 524 (10.3) | 363 (8.1) | 464 (9.7) | 429 (9.5) | 439 (10.3) | 4025 (9.3) |

**Table S2**. Prevalence of elevated waist-to-height ratio (WHtR) in American adults, 1999-2018

| Group (%) | 1999-2000 | 2001-2002 | 2003-2004 | 2005-2006 | 2007-2008 | 2009-2010 | 2011-2012 | 2013-2014 | 2015-2016 | 2017-2018 |
| --- | --- | --- | --- | --- | --- | --- | --- | --- | --- | --- |
| Overall | 74.8 (72.4-77.3) | 75.5 (74.1-76.8) | 79.1 (77.1-81.1) | 77.9 (75.3-80.5) | 79.3 (77.9-80.7) | 79.7 (76.7-82.8) | 80.9 (77.6-84.2) | 82.3 (80.7-83.9) | 83.6 (81.5-85.6) | 82.7 (80.1-85.2) |
| Sex |  |  |  |  |  |  |  |  |  |  |
| Men | 76.8 (73.8-79.8) | 78.0 (75.7-80.3) | 80.3 (78.1-82.5) | 80.5 (77.5-83.6) | 80.0 (78.5-81.5) | 80.5 (76.7-84.3) | 80.2 (76.6-83.7) | 82.0 (80.0-83.9) | 82.5 (80.4-84.7) | 83.1 (80.2-86.0) |
| Women | 72.9 (69.6-76.3) | 73.1 (71.1-75.0) | 77.9 (74.8-81.1) | 75.4 (72.4-78.5) | 78.6 (76.1-81.0) | 79.0 (76.0-82.0) | 81.7 (78.2-85.1) | 82.6 (80.4-84.9) | 84.6 (81.8-87.4) | 72.9 (69.6-76.3) |
| Ethnicity |  |  |  |  |  |  |  |  |  |  |
| Mexican American | 84.9 (81.8-88.0) | 82.0 (78.6-85.4) | 86.9 (82.3-91.5) | 87.5 (84.3-90.7) | 90.0 (87.8-92.3) | 90.3 (88.1-92.4) | 89.3 (84.9-93.7) | 90.8 (88.1-93.5) | 92.4 (90.7-94.2) | 91.6 (87.6-95.6) |
| Other Hispanic | 79.4 (73.0-85.8) | 79.7 (70.8-88.6) | 81.5 (74.9-88.1) | 84.7 (76.5-92.9) | 84.7 (80.3-89.1) | 82.7 (80.9-84.5) | 86.7 (82.7-90.6) | 84.0 (78.9-89.1) | 87.5 (84.3-90.8) | 86.5 (82.8-90.2) |
| Non-Hispanic White | 73.4 (70.2-76.6) | 75.2 (73.6-76.8) | 78.5 (75.8-81.2) | 77.1 (73.9-80.2) | 78.7 (76.6-80.8) | 79.3 (75.5-83.0) | 80.9 (77.2-84.6) | 82.2 (80.3-84.1) | 83.2 (80.9-85.6) | 82.0 (78.9-85.2) |
| Non-Hispanic Black | 73.4 (69.9-76.9) | 72.0 (68.0-75.9) | 78.9 (76.1-81.8) | 77.5 (74.3-80.8) | 77.5 (75.3-79.6) | 78.6 (74.6-82.6) | 79.5 (76.8-82.3) | 80.5 (78.3-82.8) | 79.8 (76.6-83.0) | 78.7 (75.7-81.7) |
| Other race | 76.1 (62.5-89.8) | 72.5 (65.9-79.1) | 74.5 (67.6-81.4) | 72.4 (63.4-81.3) | 69.9 (63.8-75.9) | 70.6 (61.2-79.9) | 69.9 (63.2-76.6) | 74.7 (69.7-79.7) | 79.0 (74.4-83.6) | 81.1 (77.8-84.3) |
| Age group |  |  |  |  |  |  |  |  |  |  |
| 20-39 years old | 62.3 (58.7-65.9) | 63.4 (59.9-66.9) | 65.7 (62.5-68.8) | 65.9 (61.4-70.3) | 65.7 (62.6-68.8) | 67.7 (62.6-72.9) | 65.8 (60.5-71.1) | 70.0 (66.8-73.3) | 69.7 (66.4-72.9) | 69.9 (64.5-75.3) |
| 40-59 years old | 78.6 (74.6-82.6) | 79.1 (76.5-81.7) | 84.8 (81.7-87.8) | 82.7 (80.2-85.3) | 84.0 (81.7-86.3) | 82.9 (79.8-86.0) | 87.7 (85.5-89.9) | 86.5 (83.9-89.0) | 89.3 (86.9-91.7) | 86.6 (83.9-89.2) |
| ≥60 years old | 90.6 (88.7-92.5) | 90.4 (88.8-92.0) | 92.1 (90.3-94.0) | 89.2 (87.4-91.0) | 93.0 (91.2-94.7) | 92.6 (91.3-93.9) | 92.3 (88.5-96.1) | 93.4 (91.4-95.4) | 93.9 (92.3-95.5) | 93.7 (92.3-95.1) |
| Smoking status |  |  |  |  |  |  |  |  |  |  |
| Never smoker | 73.4 (69.7-77.2) | 74.8 (72.4-77.3) | 77.6 (74.6-80.6) | 77.7 (74.5-81.0) | 79.1 (76.7-81.5) | 79.6 (75.7-83.5) | 79.5 (75.0-83.9) | 81.3 (78.7-84.0) | 81.3 (78.5-84.1) | 81.2 (78.1-84.3) |
| Former smoker | 85.3 (82.9-87.8) | 82.1 (79.6-84.5) | 86.4 (83.8-89.1) | 85.1 (82.4-87.8) | 86.4 (83.3-89.6) | 85.1 (81.4-88.7) | 87.9 (85.2-90.6) | 89.7 (87.8-91.6) | 88.2 (85.4-91.0) | 89.9 (86.5-93.3) |
| Current smoker | 67.0 (62.6-71.4) | 70.0 (67.0-72.9) | 74.9 (71.9-77.8) | 71.2 (67.6-74.7) | 72.1 (68.4-75.7) | 73.7 (70.3-77.0) | 76.7 (73.5-79.9) | 76.3 (73.3-79.2) | 84.2 (80.9-87.4) | 77.2 (72.7-81.7) |
| Education level |  |  |  |  |  |  |  |  |  |  |
| Less than high school | 82.8 (79.1-86.5) | 81.7 (78.4-85.1) | 83.1 (79.4-86.8) | 83.7 (81.2-86.2) | 82.8 (80.3-85.3) | 85.9 (81.5-90.3) | 88.7 (86.7-90.7) | 85.6 (82.2-89.0) | 89.6 (87.1-92.1) | 85.9 (81.5-90.2) |
| High school graduate | 75.7 (72.4-78.9) | 78.2 (75.1-81.4) | 83.7 (80.3-87.0) | 80.8 (78.2-83.4) | 81.8 (79.4-84.2) | 80.7 (77.1-84.3) | 86.0 (83.5-88.6) | 84.3 (81.9-86.8) | 88.1 (85.2-91.0) | 84.7 (81.6-87.9) |
| College or above | 70.3 (66.5-74.1) | 72.1 (70.7-73.4) | 75.6 (73.0-78.2) | 75.0 (71.5-78.5) | 76.8 (74.8-78.8) | 77.3 (72.5-82.2) | 77.3 (72.7-81.8) | 80.8 (78.8-82.9) | 80.8 (78.3-83.4) | 81.2 (77.8-84.6) |
| Poverty income ratio |  |  |  |  |  |  |  |  |  |  |
| In poverty (< 1.00) | 73.8 (69.2-78.4) | 74.2 (71.2-77.3) | 78.4 (73.9-82.8) | 79.5 (76.4-82.6) | 75.3 (70.2-80.4) | 77.8 (72.0-83.6) | 77.8 (69.8-85.8) | 77.3 (73.8-80.8) | 85.1 (81.0-89.2) | 80.9 (76.2-85.7) |
| Not in poverty (≥ 1.00) | 75.3 (72.2-78.3) | 75.4 (73.9-77.0) | 79.4 (77.3-81.5) | 77.8 (74.9-80.6) | 80.0 (78.6-81.4) | 80.0 (76.7-83.2) | 81.4 (78.0-84.8) | 83.4 (81.4-85.3) | 83.3 (80.9-85.6) | 82.7 (79.9-85.5) |
| Diabetes |  |  |  |  |  |  |  |  |  |  |
| Yes | 97.3 (95.0-99.6) | 94.2 (91.5-96.9) | 94.9 (92.6-97.3) | 94.3 (92.2-96.3) | 96.1 (94.0-98.2) | 96.5 (94.8-98.1) | 97.2 (95.8-98.7) | 98.4 (97.7-99.0) | 96.7 (94.3-99.0) | 97.7 (96.2-99.2) |
| No | 72.9 (70.4-75.4) | 73.8 (72.4-75.3) | 77.3 (75.0-79.6) | 75.9 (73.0-78.8) | 76.8 (75.1-78.5) | 77.2 (73.6-80.8) | 78.4 (74.9-82.0) | 79.8 (77.9-81.6) | 81.2 (79.1-83.2) | 80.0 (77.0-83.0) |
| Chronic kidney disease |  |  |  |  |  |  |  |  |  |  |
| Yes | 83.4 (76.9-89.8) | 82.6 (77.6-87.6) | 85.6 (78.9-92.3) | 84.5 (81.1-87.8) | 86.6 (83.0-90.3) | 84.9 (80.1-89.7) | 85.3 (79.4-91.2) | 87.9 (84.4-91.5) | 88.6 (84.7-92.4) | 88.7 (85.0-92.5) |
| No | 73.8 (71.4-76.3) | 74.7 (73.2-76.1) | 78.8 (76.7-80.8) | 77.4 (74.5-80.3) | 78.7 (77.2-80.1) | 79.5 (76.4-82.5) | 80.5 (77.2-83.8) | 81.7 (80.1-83.3) | 83.0 (80.7-85.3) | 82.0 (79.2-84.9) |
| Hypertension |  |  |  |  |  |  |  |  |  |  |
| Yes | 91.3 (89.4-93.2) | 88.1 (86.1-90.2) | 90.6 (88.6-92.5) | 88.9 (87.0-90.8) | 91.5 (90.5-92.5) | 92.8 (91.2-94.5) | 92.8 (90.7-94.9) | 93.0 (91.2-94.8) | 94.8 (93.4-96.1) | 93.4 (91.9-94.8) |
| No | 66.3 (63.4-69.2) | 68.9 (67.2-70.7) | 72.4 (70.0-74.7) | 71.7 (68.2-75.2) | 72.2 (70.0-74.5) | 72.6 (68.6-76.6) | 74.0 (69.9-78.1) | 75.5 (73.1-77.8) | 76.7 (73.8-79.6) | 75.5 (72.2-78.9) |
| Cardiovascular disease |  |  |  |  |  |  |  |  |  |  |
| Yes | 90.4 (86.2-94.7) | 91.2 (88.3-94.2) | 92.9 (90.3-95.5) | 91.5 (89.2-93.7) | 93.6 (90.1-97.0) | 93.4 (91.3-95.5) | 94.3 (91.9-96.6) | 92.3 (89.7-94.9) | 95.4 (92.9-98.0) | 94.6 (91.7-97.4) |
| No | 73.4 (70.8-76.0) | 74.1 (72.8-75.5) | 77.7 (75.5-79.9) | 76.7 (74.0-79.5) | 78.0 (76.6-79.4) | 78.6 (75.4-81.8) | 79.8 (76.3-83.2) | 81.4 (79.6-83.2) | 82.5 (80.4-84.7) | 81.5 (78.7-84.2) |
| Cancer |  |  |  |  |  |  |  |  |  |  |
| Yes | 76.8 (69.6-83.9) | 85.6 (82.2-89.0) | 84.7 (80.8-88.7) | 81.8 (76.8-86.8) | 86.9 (84.4-89.3) | 87.8 (84.6-91.1) | 87.4 (83.6-91.2) | 90.7 (87.5-93.9) | 92.4 (90.2-94.5) | 89.3 (84.6-94.0) |
| No | 74.7 (72.0-77.4) | 74.5 (73.1-75.9) | 78.6 (76.4-80.8) | 77.6 (74.9-80.3) | 78.5 (76.9-80.1) | 78.8 (75.5-82.1) | 80.3 (76.8-83.7) | 81.3 (79.5-83.0) | 82.5 (80.3-84.7) | 81.9 (79.2-84.6) |

*Central obesity is defined as having a waist-to-height ratio≥0.5.

**Table S3**. Prevalence of elevated waist circumference (WC) in American adults, 1999-2018

| Group (%) | 1999-2000 | 2001-2002 | 2003-2004 | 2005-2006 | 2007-2008 | 2009-2010 | 2011-2012 | 2013-2014 | 2015-2016 | 2017-2018 |
| --- | --- | --- | --- | --- | --- | --- | --- | --- | --- | --- |
| Overall | 46.9 (42.6-51.2) | 48.5 (46.7-50.4) | 52.8 (50.3-55.3) | 52.7 (49.1-56.2) | 53.3 (50.6-56.0) | 53.9 (51.1-56.8) | 55.3 (51.6-59.1) | 57.0 (55.8-58.3) | 59.4 (55.1-63.7) | 60.3 (57.0-63.6) |
| Sex |  |  |  |  |  |  |  |  |  |  |
| Men | 36.8 (32.8-40.7) | 39.1 (37.1-41.0) | 42.8 (40.4-45.1) | 45.4 (40.8-49.9) | 43.6 (40.4-46.8) | 43.5 (40.0-47.1) | 44.3 (40.6-47.9) | 46.1 (44.0-48.2) | 48.3 (43.1-53.5) | 51.4 (47.0-55.8) |
| Women | 56.7 (51.8-61.6) | 57.5 (54.8-60.3) | 62.4 (58.9-65.9) | 59.7 (56.3-63.1) | 62.5 (59.5-65.6) | 64.0 (61.4-66.6) | 66.1 (62.0-70.2) | 67.5 (65.1-70.0) | 70.1 (65.9-74.3) | 68.8 (65.1-72.5) |
| Ethnicity |  |  |  |  |  |  |  |  |  |  |
| Mexican American | 44.5 (41.3-47.7) | 42.9 (39.8-46.0) | 50.9 (43.6-58.2) | 47.5 (45.1-49.8) | 52.2 (44.8-59.7) | 55.7 (51.4-59.9) | 55.9 (51.2-60.7) | 62.3 (57.8-66.8) | 64.2 (60.9-67.4) | 65.5 (61.0-70.0) |
| Other Hispanic | 42.9 (36.9-49.0) | 45.3 (36.7-53.9) | 49.0 (38.8-59.1) | 51.3 (43.0-59.6) | 52.5 (46.8-58.2) | 48.9 (42.4-55.5) | 56.0 (50.5-61.5) | 53.2 (46.6-59.7) | 55.5 (50.2-60.9) | 50.5 (45.4-55.6) |
| Non-Hispanic White | 47.2 (41.8-52.5) | 50.1 (48.1-52.1) | 53.8 (50.3-57.3) | 53.6 (49.6-57.5) | 55.1 (51.6-58.5) | 55.1 (52.0-58.3) | 56.8 (52.2-61.4) | 58.5 (56.8-60.3) | 61.9 (56.9-66.8) | 62.4 (58.1-66.7) |
| Non-Hispanic Black | 52.4 (49.5-55.3) | 49.7 (46.4-53.0) | 56.6 (53.9-59.4) | 56.4 (53.4-59.4) | 56.3 (53.0-59.6) | 59.8 (54.5-65.1) | 60.6 (57.4-63.8) | 61.9 (58.4-65.4) | 58.7 (54.4-63.1) | 61.9 (58.8-65.1) |
| Other race | 40.3 (26.8-53.8) | 32.1 (23.6-40.6) | 36.5 (27.8-45.2) | 41.0 (33.3-48.7) | 29.3 (22.4-36.3) | 34.2 (28.2-40.2) | 34.4 (27.4-41.4) | 34.4 (29.5-39.2) | 41.1 (33.9-48.3) | 47.9 (43.1-52.7) |
| Age group |  |  |  |  |  |  |  |  |  |  |
| 20-39 years old | 34.2 (29.8-38.5) | 35.7 (32.9-38.5) | 38.3 (35.1-41.6) | 37.7 (33.4-42.1) | 39.7 (35.6-43.8) | 40.8 (36.2-45.4) | 40.9 (36.3-45.4) | 44.7 (41.8-47.5) | 46.5 (42.6-50.4) | 47.3 (42.1-52.4) |
| 40-59 years old | 51.4 (45.6-57.3) | 51.8 (48.3-55.3) | 58.4 (55.5-61.3) | 58.3 (53.8-62.7) | 57.6 (53.9-61.4) | 57.3 (54.2-60.4) | 60.8 (57.3-64.2) | 59.8 (56.9-62.8) | 63.2 (57.8-68.6) | 63.5 (59.3-67.7) |
| ≥60 years old | 61.8 (57.5-66.1) | 65.7 (62.9-68.6) | 67.7 (64.5-71.0) | 67.2 (64.2-70.3) | 67.9 (64.9-70.9) | 68.3 (65.4-71.2) | 67.9 (63.5-72.2) | 70.2 (66.4-74.1) | 71.2 (66.0-76.3) | 72.5 (68.7-76.3) |
| Smoking status |  |  |  |  |  |  |  |  |  |  |
| Never smoker | 47.1 (41.9-52.2) | 48.4 (45.6-51.1) | 53.3 (49.1-57.4) | 53.4 (50.0-56.7) | 52.6 (48.9-56.3) | 53.5 (49.3-57.7) | 54.5 (50.2-58.8) | 55.4 (52.5-58.3) | 57.4 (52.7-62.2) | 59.3 (55.7-62.8) |
| Former smoker | 54.4 (48.7-60.1) | 54.5 (50.9-58.1) | 59.9 (56.4-63.4) | 58.9 (54.2-63.5) | 61.7 (59.1-64.3) | 60.9 (57.1-64.6) | 63.4 (58.4-68.4) | 63.1 (59.2-67.1) | 67.4 (61.4-73.4) | 66.3 (61.0-71.5) |
| Current smoker | 38.8 (33.7-44.0) | 42.8 (40.0-45.6) | 45.0 (41.6-48.5) | 44.9 (39.9-50.0) | 46.2 (42.1-50.2) | 46.8 (43.0-50.6) | 48.0 (44.6-51.4) | 54.4 (51.8-57.0) | 54.5 (49.2-59.9) | 55.1 (51.3-58.8) |
| Education level |  |  |  |  |  |  |  |  |  |  |
| Less than high school | 54.1 (49.7-58.5) | 53.0 (50.5-55.4) | 54.3 (50.2-58.4) | 55.0 (51.9-58.2) | 55.0 (51.0-59.0) | 59.0 (54.3-63.7) | 58.2 (55.7-60.7) | 59.0 (56.1-61.8) | 57.8 (53.9-61.7) | 58.7 (54.5-62.8) |
| High school graduate | 50.1 (46.2-53.9) | 50.3 (45.8-54.8) | 57.9 (54.5-61.2) | 56.8 (51.1-62.5) | 56.3 (52.4-60.1) | 56.8 (53.8-59.8) | 61.3 (55.2-67.4) | 61.7 (58.8-64.6) | 64.2 (59.2-69.2) | 64.2 (60.4-67.9) |
| College or above | 41.5 (35.7-47.3) | 46.2 (43.5-48.9) | 49.8 (46.1-53.6) | 50.2 (46.3-54.1) | 51.3 (48.1-54.6) | 51.2 (47.3-55.1) | 52.7 (48.2-57.3) | 55.0 (52.8-57.1) | 58.2 (53.1-63.4) | 58.9 (54.7-63.1) |
| Poverty income ratio |  |  |  |  |  |  |  |  |  |  |
| In poverty (< 1.00) | 51.5 (46.8-56.3) | 48.5 (42.6-54.4) | 51.9 (47.4-56.4) | 49.7 (45.2-54.3) | 51.4 (45.4-57.4) | 54.5 (49.1-60.0) | 52.5 (45.9-59.2) | 57.4 (53.8-61.1) | 61.4 (56.2-66.7) | 60.0 (55.1-64.9) |
| Not in poverty (≥ 1.00) | 46.4 (41.3-51.4) | 48.0 (46.0-50.0) | 53.0 (50.4-55.6) | 53.1 (49.1-57.0) | 53.6 (51.0-56.3) | 54.2 (51.3-57.1) | 55.9 (51.8-60.0) | 57.2 (55.7-58.6) | 59.7 (55.1-64.3) | 60.5 (57.2-63.8) |
| Diabetes |  |  |  |  |  |  |  |  |  |  |
| Yes | 78.9 (73.1-84.7) | 74.1 (68.8-79.4) | 79.6 (74.4-84.9) | 81.5 (79.1-83.9) | 79.8 (76.3-83.3) | 81.0 (76.2-85.8) | 81.7 (78.3-85.0) | 83.3 (80.5-86.1) | 80.7 (76.2-85.2) | 83.0 (78.8-87.2) |
| No | 44.1 (39.9-48.3) | 46.3 (44.5-48.1) | 49.7 (47.1-52.3) | 49.1 (45.4-52.8) | 49.4 (46.5-52.3) | 49.8 (46.9-52.8) | 51.3 (47.5-55.2) | 52.9 (51.5-54.3) | 55.5 (51.2-59.8) | 56.3 (52.6-59.9) |
| Chronic kidney disease |  |  |  |  |  |  |  |  |  |  |
| Yes | 60.1 (52.0-68.2) | 62.3 (56.5-68.0) | 63.2 (56.1-70.2) | 65.8 (62.5-69.1) | 66.1 (61.0-71.2) | 65.7 (60.9-70.6) | 66.6 (61.1-72.1) | 68.5 (64.4-72.6) | 66.0 (61.7-70.2) | 73.8 (68.2-79.3) |
| No | 45.2 (41.0-49.5) | 47.1 (45.2-49.1) | 52.0 (49.3-54.8) | 51.4 (47.7-55.2) | 52.0 (49.3-54.7) | 53.1 (50.2-55.9) | 54.2 (50.5-58.0) | 55.9 (54.6-57.2) | 58.7 (54.2-63.2) | 58.9 (55.2-62.5) |
| Hypertension |  |  |  |  |  |  |  |  |  |  |
| Yes | 67.3 (63.6-71.0) | 65.5 (62.5-68.6) | 69.9 (66.6-73.3) | 68.8 (66.0-71.7) | 69.4 (66.6-72.2) | 72.9 (70.5-75.3) | 70.8 (67.3-74.3) | 72.3 (70.3-74.2) | 73.7 (69.3-78.1) | 75.4 (72.5-78.3) |
| No | 36.3 (32.1-40.5) | 39.8 (38.1-41.5) | 42.8 (40.6-45.0) | 43.5 (39.5-47.5) | 44.1 (41.2-47.0) | 43.7 (40.2-47.1) | 46.4 (42.4-50.4) | 47.3 (45.0-49.5) | 50.7 (46.1-55.4) | 50.2 (46.8-53.6) |
| Cardiovascular disease |  |  |  |  |  |  |  |  |  |  |
| Yes | 61.4 (56.3-66.5) | 65.6 (59.2-72.0) | 69.7 (64.8-74.5) | 77.6 (73.8-81.4) | 71.5 (66.1-76.9) | 71.5 (67.4-75.6) | 64.6 (58.4-70.8) | 72.5 (68.2-76.9) | 73.5 (67.1-79.9) | 73.8 (66.5-81.2) |
| No | 45.5 (41.2-49.9) | 47.1 (45.2-49.0) | 51.1 (48.6-53.6) | 50.4 (46.8-54.0) | 51.7 (49.0-54.4) | 52.5 (49.4-55.6) | 54.6 (50.5-58.6) | 55.6 (54.0-57.2) | 58.2 (53.8-62.6) | 58.9 (55.7-62.2) |
| Cancer |  |  |  |  |  |  |  |  |  |  |
| Yes | 48.2 (38.5-57.9) | 62.6 (59.1-66.0) | 63.8 (58.9-68.6) | 62.4 (54.4-70.4) | 66.4 (61.4-71.5) | 64.8 (58.8-70.9) | 65.2 (61.0-69.4) | 65.8 (61.5-70.1) | 69.7 (64.3-75.0) | 71.4 (66.7-76.0) |
| No | 46.8 (42.5-51.1) | 47.2 (45.1-49.2) | 51.7 (49.0-54.5) | 51.8 (48.0-55.6) | 51.9 (49.4-54.5) | 52.7 (49.8-55.6) | 54.4 (50.4-58.4) | 55.9 (54.5-57.4) | 58.2 (53.7-62.7) | 58.9 (55.5-62.4) |

Central obesity is defined as have a waist circumference≥102 cm for male or ≥88 cm for female.

**Table S4.** Pairwise comparison of trend central obesity categorized by sociodemographic characteristics and co-morbidities

| Group 1 | Group 2 | P-value for coincidence |
| --- | --- | --- |
| **Waist-to-height ratio** |  |  |
| Men | Women | 0.010 |
| Mexican American | Other Hispanic | 0.001 |
| Mexican American | Non-Hispanic White | <0.001 |
| Mexican American | Non-Hispanic Black | <0.001 |
| Mexican American | Other race | <0.001 |
| Other Hispanic | Non-Hispanic White | <0.001 |
| Other Hispanic | Non-Hispanic Black | <0.001 |
| Other Hispanic | Other race | 0.001 |
| Non-Hispanic White | Non-Hispanic Black | 0.041 |
| Non-Hispanic White | Other race | <0.001 |
| Non-Hispanic Black | Other race | 0.008 |
| 20-39 years old | 40-59 years old | 0.001 |
| 20-39 years old | ≥60 years old | 0.001 |
| 40-59 years old | ≥60 years old | 0.001 |
| Never smoker | Former smoker | <0.001 |
| Never smoker | Current smoker | 0.036 |
| Former smoker | Current smoker | 0.001 |
| Less than high school | High school graduate | 0.003 |
| Less than high school | College or above | <0.001 |
| High school graduate | College or above | 0.001 |
| Not in poverty | In poverty | 0.163 |
| Diabetes | Without diabetes | <0.001 |
| Chronic kidney disease | Without chronic kidney disease | <0.001 |
| Hypertension | Without hypertension | 0.001 |
| Cardiovascular disease | Without cardiovascular disease | <0.001 |
| Cancer | Without cancer | 0.001 |
|  |  |  |
| **Waist circumference** |  |  |
| Men | Women | <0.001 |
| Mexican American | Other Hispanic | 0.078 |
| Mexican American | Non-Hispanic White | 0.005 |
| Mexican American | Non-Hispanic Black | 0.003 |
| Mexican American | Other race | 0.001 |
| Other Hispanic | Non-Hispanic White | 0.001 |
| Other Hispanic | Non-Hispanic Black | 0.002 |
| Other Hispanic | Other race | <0.001 |
| Non-Hispanic White | Non-Hispanic Black | 0.006 |
| Non-Hispanic White | Other race | <0.001 |
| Non-Hispanic Black | Other race | <0.001 |
| 20-39 years old | 40-59 years old | 0.001 |
| 20-39 years old | ≥60 years old | 0.001 |
| 40-59 years old | ≥60 years old | 0.001 |
| Never smoker | Former smoker | 0.001 |
| Never smoker | Current smoker | 0.001 |
| Former smoker | Current smoker | 0.001 |
| Less than high school | High school graduate | 0.096 |
| Less than high school | College or above | 0.003 |
| High school graduate | College or above | <0.001 |
| In poverty | Not in poverty | 0.021 |
| Diabetes | Without diabetes | 0.001 |
| Chronic kidney disease | Without chronic kidney disease | <0.001 |
| Hypertension | Without hypertension | 0.001 |
| Cardiovascular disease | Without cardiovascular disease | 0.001 |
| Cancer | Without cancer | 0.001 |

**Table S5.** Proportions of central obesity subgroups in American adults, 1999-2018

| Group (%) | 1999-2000 | 2001-2002 | 2003-2004 | 2005-2006 | 2007-2008 | 2009-2010 | 2011-2012 | 2013-2014 | 2015-2016 | 2017-2018 | Overall |
| --- | --- | --- | --- | --- | --- | --- | --- | --- | --- | --- | --- |
| Normal WC, normal WHtR | 25.1  (22.7-27.5) | 24.4  (23.0-25.7) | 20.9  (18.9-22.8) | 22.1  (19.4-24.7) | 20.6  (19.1-22.1) | 20.2  (17.2-23.2) | 19.1  (15.8-22.4) | 17.6  (16.0-19.3) | 16.4  (14.4-18.5) | 17.3  (14.8-19.8) | 20.2  (19.5-20.9) |
| Normal WC, elevated WHtR | 28.0  (25.3-30.7) | 27.1  (25.5-28.6) | 26.4  (24.7-28.0) | 25.3  (23.8-26.8) | 26.1  (23.7-28.4) | 25.8  (24.3-27.4) | 25.6  (23.7-27.5) | 25.3  (24.1-26.6) | 24.2  (21.3-27.0) | 22.4  (20.6-24.2) | 25.5  (24.9-26.1) |
| Elevated WC, normal WHtR | 0.1  (0.0-0.2) | 0.2  (0.0-0.3) | 0.0  (0.0-0.1) | 0.0  (0.0-0.0) | 0.1  (0.0-0.3) | 0.1  (0.0-0.1) | 0.0  (0.0-0.0) | 0.1  (-0.1-0.2) | 0.0  (0.0-0.0) | 0.0  (0.0-0.0) | 0.1  (0.0-0.1) |
| Elevated WC, elevated WHtR | 46.8  (42.5-51.1) | 48.4  (46.6-50.2) | 52.8  (50.2-55.3) | 52.7  (49.1-56.2) | 53.2  (50.5-55.9) | 53.9  (51.0-56.7) | 55.3  (51.6-59.1) | 57.0  (55.7-58.2) | 59.4  (55.1-63.7) | 60.3  (57.0-63.5) | 54.2  (53.2-55.2) |

Abbreviations: WHtR, waist-to-height ratio; WC, waist circumference

Normal level of WHtR is defined as <0.5. Normal level of WC is defined as <102cm for male or <88cm for female.

Elevated level of WHtR is defined as ≥0.5. Elevated level of WC is defined as ≥102cm for male or ≥88cm for female.

1. Each NHANES participant may have missing values for more than one variable. Thus, the total number of excluded participants for association analyses does not equal to the sum of missing counts of each variable. [↑](#footnote-ref-1)
